# Higher serum Lp-PLA2 is associated with cognitive impairment in Parkinson’s disease patients

**DOI:** 10.3389/fnins.2024.1374567

**Published:** 2024-03-12

**Authors:** Zubo Wu, Defeng Shu, Suyuan Wu, Pengcheng Cai, Tao Liang

**Affiliations:** ^1^Department of Pediatrics, Union Hospital, Tongji Medical College, Huazhong University of Science and Technology, Wuhan, China; ^2^Department of Obstetrics and Gynecology, Union Hospital, Tongji Medical College, Huazhong University of Science and Technology, Wuhan, China; ^3^Department of Clinical Laboratory, Union Hospital, Tongji Medical College, Huazhong University of Science and Technology, Wuhan, China

**Keywords:** Lp-PLA2, Parkinson’s disease, cognitive impairment, risk factor, neurodegenerative diseases

## Abstract

**Objective:**

To explore the association between lipoprotein-associated phospholipase A2 (Lp-PLA2) and the risk of cognitive impairment in Parkinson’s disease (PD-CI).

**Methods:**

A case–control study involving 100 hospitalized PD patients and 60 healthy controls was carried out. Serum Lp-PLA2 level was detected by automatic biochemical analyzer. Based on whether Parkinson’s patients have cognitive impairment, PD patients were subdivided to analyze the clinical value of Lp-PLA2. Relationship between Lp-PLA2 and PD-CI risk was analyzed by logistic regression. Diagnostic value of Lp-PLA2 in PD-CI patients was investigated using receiver’s operator characteristic curves.

**Results:**

The levels of serum Lp-PLA2 activity in Parkinson’s disease with normal cognition (PD-NC) and PD-CI patients were significantly higher than those in healthy controls (HCs), respectively. Furthermore, compared to the PD-NC group, the serum Lp-PLA2 activity level was significantly higher in PD-CI patients. Multivariable logistic regression analysis indicated that higher Lp-PLA2 level was an independent risk factor for PD patients with cognitive impairment. Moreover, the area under the efficacy curve of Lp-PLA2 for predicting PD-CI is 0.659.

**Conclusion:**

Our study shows that higher levels of Lp-PLA2 activity in PD patients are associated with the risk of developing cognitive impairment. Therefore, given the wide availability, safety, and convenience of monitoring serum Lp-PLA2 activity, it may serve as an early biomarker for cognitive impairment in PD patients.

## 1 Introduction

Parkinson’s disease (PD) is one of the most common neurodegenerative disorders affecting individuals worldwide (Aarsland et al., 2021). It is characterized by a progressive loss of dopaminergic neurons in the substantia nigra region of the brain leading to motor symptoms that include tremors, rigidity, and bradykinesia. PD has been identified as a multifactorial disorder with both genetic and environmental factors contributing to its pathogenesis (Bloem et al., 2021). In addition to motor symptoms, PD also has a variety of non-motor symptoms. Among them, the most common is cognitive impairment, which can occur in any disease stage of PD and seriously affect the patient’s quality of life. In recent years, there has been growing interest in the relationship between PD and cognitive impairment (Aarsland et al., 2021). Cognitive impairment refers to a decline in intellectual and cognitive functions, including memory loss and attention deficits (Weintraub et al., 2022). Depending on the severity, cognitive impairment can be divided into mild cognitive impairment (MCI) and Parkinson’s disease dementia (PDD). In most cases, cognitive decline is usually slow and insidious. In recent years, many studies on PD and cognitive impairment have focused on early cognitive changes, especially actively screening for better biomarkers to predict cognitive decline and quickly identify patients at risk of early cognitive impairment (Aarsland et al., 2021). Recent research indicates that a substantial proportion, approximately 30–40%, of individuals with Parkinson’s disease (PD) encounter mild cognitive impairment, with a subset of 10–20% advancing to Parkinson’s disease-related cognitive impairment (PD-CI) (Aarsland et al., 2021; Wallace et al., 2022). Investigations have proposed a potential correlation between PD-CI and the inherent pathological transformations in PD, as well as modifications in neurotransmitter activity (Zaman et al., 2021; Ye et al., 2023).

Lipoprotein-associated phospholipase A2 (Lp-PLA2) is an enzyme synthesized by macrophages, monocytes, and other inflammatory cells, including endothelial cells in atherosclerotic plaques. It is also found in circulating low-density lipoprotein (LDL) particles. Lp-PLA2 catalyzes the hydrolysis of oxidized phospholipids (OxPLs) which can result in the generation of pro-inflammatory and pro-apoptotic products (Huang et al., 2020). Lp-PLA2 levels have been associated with the severity of atherosclerosis, and high levels of Lp-PLA2 are considered a risk factor for cardiovascular disease (Ridker et al., 2022).

Recent research has suggested that Lp-PLA2 may also be involved in the pathogenesis of cognitive impairment. In one study, Lp-PLA2 levels were found to be higher in individuals with mild cognitive impairment (MCI) and Alzheimer’s disease (AD) compared to controls (Davidson et al., 2012; Pokharel et al., 2019; Liu et al., 2023). Another study found that Lp-PLA2 levels were elevated in individuals with type 2 diabetes and that Lp-PLA2 levels were higher in diabetic individuals with cognitive impairment compared to those without cognitive impairment (Cai et al., 2017). Studies have reported the role of Lp-PLA2 in cognitive impairment-related diseases such as Alzheimer’s disease and diabetes (Davidson et al., 2012; Feng et al., 2022). These results have shown elevated plasma levels of Lp-PLA2 in patients with these diseases, which are strongly associated with cognitive decline. We have also found in previous studies that serum Lp-PLA2 levels are significantly higher in PD patients compared to healthy individuals, and that Lp-PLA2 levels are positively correlated with disease severity (Wu et al., 2021). However, the relationship between Lp-PLA2 and cognitive impairment associated with PD is unclear. Given the important role of Lp-PLA2 in neurodegenerative diseases such as Alzheimer’s disease, it is suspected that they may also play significant roles in PD-CI.

In conclusion, recent research suggests that Lp-PLA2 may play an important role in the development and progression of PD and cognitive impairments. Further investigation is required to determine the exact mechanisms by which Lp-PLA2 contributes to the development of cognitive impairment in PD, and whether Lp-PLA2 could be a potential therapeutic target for the treatment of cognitive impairment in PD.

## 2 Subjects and methods

### 2.1 Subjects

A total of 100 hospitalized PD patients from the Department of Neurology, Tongji Medical College Affiliated Union Hospital, Huazhong University of Science and Technology between June 2022 and March 2023 were recruited. There were 58 males and 42 females, and they were aged from 31 to 85 years, with an average of 62.28 ± 11.51 years. All cases were diagnosed by specialists according to typical clinical symptoms and imaging examinations, and in accordance with the diagnostic criteria of PD in the UK Brain Bank (Hughes et al., 2001). The exclusion criteria are as follows: (1) Parkinson’s symptoms caused by brain trauma, encephalitis and drugs; (2) essential tremor; (3) PD with tumors, severe infection of the whole body or CNS; (4) PD with severe diseases of the heart, liver and kidney. In addition, a Montreal Cognitive Assessment (MoCA) scale was performed on PD patients to assess their cognitive status. MoCA is mainly based on clinical experience and reference to the cognitive items in the Simple Mental State Assessment Scale (MMSE). The test time is short, and the total score is 0–30 points. The cut-off value is 26, that is, ≥26 is classified as normal cognitive function, <26 is classified as cognitive impairment. According to the MoCA points, PD patients were divided into a PD-NC group (*n* = 46) and a PD-CI group (*n* = 54). The age- and gender-matched control group consisted of 60 healthy subjects (28 males/32 females, median age 55.97 ± 11.23 years), who were recruited from the medical examination center in our hospital and had no evidence of cerebrovascular or inflammatory disease. All subjects were Chinese Han from the same area in Middle China and all gave informed consent. Our study was approved by the ethics committee at Tongji Medical College, Huazhong University of Science and Technology.

### 2.2 Data collection

After PD patients were admitted to the hospital, their demographic data were obtained in time, including age, gender, disease course, past history, family history, and smoking and drinking habits. Clinical characteristic parameters of PD patients were also collected, such as diastolic blood pressure (DBP), systolic blood pressure (SBP), dyslipidemia, hypertension, diabetes, heart rate, respiratory rate, and body temperature. Patients with a history of hypertension or SBP≧140 mmHg or DBP≧90 mmHg at rest were all diagnosed as hypertension. Diabetes mellitus was diagnosed if the patient was being treated with antidiabetic medications or insulin therapy or had a fasting blood glucose level ≧7.0 mmol/L. We also collected other laboratory parameters upon admission, including alanine aminotransferase (ALT), aspartate aminotransferase (AST), alkaline phosphatase (ALP), glutamyl transpeptidase (GGT), urea nitrogen (BUN), creatinine (Cr), uric acid (UA), cystatin C (Cys C), fasting blood glucose (Glu), cholesterol (TC), triglycerides (TG), high-density lipoprotein (HDL), low-density lipoprotein (LDL), apolipoprotein A (ApoA), apolipoprotein B (ApoB), serum amylase A (SAA), Lipoprotein (a) [Lp(a)], homocysteine (HCY), glucose (GLU), glomerular filtration rate (GFR) and other blood coagulation related indicators.

### 2.3 Preparation of blood samples

Within 24 h of admission, blood samples were collected with BD™ yellow tubes from peripheral veins of PD patients who had fasted for 12 h. The blood samples were centrifuged at 3,000 rpm and room temperature for 10 min to separate serum. Then the serum was immediately transferred to Eppendorf tubes (EP tubes), labeled and stored in a refrigerator at −80°C until the detection of biochemical factors.

### 2.4 Measurement of Lp-PLA2

Serum Lp-PLA2 activity was determined by automatic biochemical analyzer AU5800 (Beckerman Coulter). Commercial Lp-PLA2 kits were also provided by Beijing Baiding Bioengineering Co., LTD. The method of Lp-PLA2 activity detection is to use enzymatic method. Lp-PLA2 decomposes the substrate to produce colored products, and the activity results can be obtained by continuous monitoring method. All operations were performed in accordance with the reagent instructions. Strict calibration and quality control procedures were also carried out.

### 2.5 Statistical analysis

SPSS 22.0 (IBM Co., Armonk, NY, United States) was used for statistical analysis. Normality data was expressed as mean ± standard deviation (SD), and statistically analyzed among groups by *t* test. Non-normally distributed data were expressed as [median (*P*50), 25th to 75th percentile (*P*25 ~ *P*75)] and compared among groups using *Mann–Whitney U* test. Relationship between any two variables was analyzed using Spearman correlation. The risk factors for PD-CI were determined by logistic regression analysis. After analyzing the difference between the PD-CI group and the PD-NC group, we chose to incorporate those significant indicators into the logistic regression analysis model. The diagnostic value of Lp-PLA2 for PD-CI was evaluated by calculating the area under receiver’s operator characteristic (ROC) curve (AUC). In the two-tailed test, *p* < 0.05 was considered statistically significant.

## 3 Results

### 3.1 Demographic and clinical characteristics of PD patients with or without cognitive impairment and healthy controls

A total of 100 PD patients and 60 healthy controls (HCs) were recruited in this study. According to the level of cognitive function, the PD patients were divided into 2 groups: 54 PD-CI patients and 46 PD-NC patients. The demographic and clinical characteristics of all participants were summarized in Table 1. The three groups were matched for sex, and there was no age difference between PD-NC and HCs. However, the age of patients in the PD-CI group was significantly higher than in the other two groups. Compared with the PD-NC group, the PD-CI patients had significantly higher H&Y stage than PD-NC group (*p* < 0.05). However, there were no significant differences between the two groups in disease course, family history, dyslipidemia, diabetes history, hypertension, smoking, drinking, BMI, etc. (all *p* > 0.05).

**Table 1:** Demographic and clinical characteristics of PD patients and HCs.

Variables	HCs (*n* = 60)	PD-NC (*n* = 46)	PD-CI (*n* = 54)	*P*-value
Gender (Male/Female)	28/32	28/18	30/24	0.592
Age (years)	57 (49 ~ 63)	57 (51 ~ 63)	71 (63 ~ 75)	<0.001
Disease duration (years)	N.A.	2 (1.0 ~ 5.0)	2 (1.0 ~ 6.0)	0.812
Family history [*n* (%)]	N.A.	3 (6.52)	3 (5.56)	0.839
Dyslipidemia [*n* (%)]	N.A.	8 (17.39)	11 (20.37)	0.705
Diabetes mellitus [*n* (%)]	N.A.	2 (4.35)	4 (7.41)	0.521
Hypertension [*n* (%)]	N.A.	17 (36.96)	20 (37.04)	0.993
Smoking [*n* (%)]	N.A.	9 (19.57)	4 (7.41)	0.072
Drinking [*n* (%)]	N.A.	5 (10.87)	1 (1.85)	0.058
SBP/ (mmHg)	N.A.	134 (122 ~ 146)	134 (123 ~ 151)	0.544
DBP/ (mmHg)	N.A.	84 (77 ~ 93)	83 (78 ~ 90)	0.579
H&Y stage	N.A.	2.00 (1.50 ~ 2.50)	2.75 (1.75 ~ 3.00)	0.019
BMI	N.A.	23.16 (21.22 ~ 24.97)	22.69 (20.60 ~ 25.68)	0.750

N.A., not available.

### 3.2 Comparison of clinical indicators among PD-NC and PD-CI patients and healthy controls

Compared with the HCs group, the levels of Cys C, HCY and Lp-PLA2 in the PD-NC group and PD-CI group were all significantly higher, while the levels of HDL and ApoA were significantly lower (*p* < 0.05). In addition, only the TG and sdLDL levels in the PD-NC group were found to be higher than that in the HCs group, but no difference was found between the PD-CI group and the HCs group (*p* > 0.05). Besides, only the Lp(a) and SAA levels in the PD-CI group were found to be higher than those in the HCs group, but no difference was found between the PD-NC group and the HCs group (*p* > 0.05). More importantly, the levels of Cys C, GFR, Lp-PLA2 were significantly increased in PD-CI patients compared with PD-NC patients (*p* < 0.05). However, there was no difference in other indicators between the two groups (*p* > 0.05) (Table 2).

**Table 2:** Comparison of clinical indicators among PD-NC and PD-CI patients and healthy controls.

Variables	HCs (Group A)	PD-NC (Group B)	PD-CI (Group C)	*P* (A vs. B)	*P* (A vs. C)	*P* (B vs. C)
UA/ (μmol/L)	275.4 (224.1 ~ 330.2)	269.5 (216.1 ~ 339.3)	297.4 (239.8 ~ 358.7)	0.975	0.489	0.511
Cys C/ (mg/L)	0.67 (0.63 ~ 0.76)	0.76 (0.66 ~ 0.81)	0.81 (0.72 ~ 0.90)	0.002	<0.001	0.010
TC/ (mmol/L)	4.53 (4.16 ~ 4.90)	4.09 (3.77 ~ 4.82)	4.15 (3.31 ~ 5.11)	0.060	0.125	0.817
TG/ (mmol/L)	0.96 (0.78 ~ 1.21)	1.10 (0.85 ~ 1.34)	0.95 (0.67 ~ 1.42)	0.049	0.966	0.167
HDL/ (mmol/L)	1.47 (1.31 ~ 1.65)	1.21 (1.03 ~ 1.43)	1.10 (0.93 ~ 1.41)	<0.001	<0.001	0.448
LDL/ (mmol/L)	2.44 (2.16 ~ 2.82)	2.38 (2.10 ~ 2.79)	2.36 (1.65 ~ 3.06)	0.975	0.986	0.977
sdLDL/ (mmol/L)	0.56 (0.41 ~ 0.78)	0.72 (0.51 ~ 0.86)	0.63 (0.46 ~ 0.92)	0.027	0.299	0.274
ApoA/ (g/L)	1.53 (1.38 ~ 1.64)	1.26 (1.12 ~ 1.40)	1.17 (1.02 ~ 1.43)	<0.001	<0.001	0.189
ApoB/ (g/L)	0.93 (0.79 ~ 1.05)	0.85 (0.77 ~ 1.00)	0.84 (0.66 ~ 1.10)	0.317	0.768	0.934
Lp(a) / (mg/dL)	11.3 (5.9 ~ 20.8)	16.0 (6.9 ~ 32.4)	17.7 (7.4 ~ 33.4)	0.110	0.026	0.559
Glu/ (mmol/L)	5.0 (4.7 ~ 5.1)	5.0 (4.6 ~ 5.2)	5.2 (4.7 ~ 5.9)	0.868	0.075	0.106
SAA/ (mg/L)	4.1 (3.1 ~ 5.8)	4.8 (3.4 ~ 6.5)	4.9 (3.6 ~ 14.6)	0.120	0.011	0.265
HCY/ (μmol/L)	10.2 (8.1 ~ 12.0)	12.5 (10.8 ~ 15.9)	13.5 (10.3 ~ 17.7)	0.001	<0.001	0.457
GFR	101.48 (94.10 ~ 108.48)	99.50 (94.38 ~ 104.25)	88.94 (81.60 ~ 99.31)	0.344	<0.001	0.001
Lp-PLA2/ (U/L)	394 (333 ~ 426)	415 (363 ~ 477)	481 (386 ~ 547)	0.019	<0.001	0.006

PD, Parkinson’s disease; HCs, healthy controls; PD-NC, PD with normal cognition; PD-CI, PD with cognitive impairment.

### 3.3 The levels of serum Lp-PLA2 activity in PD patients and healthy controls

**Figure 1:**
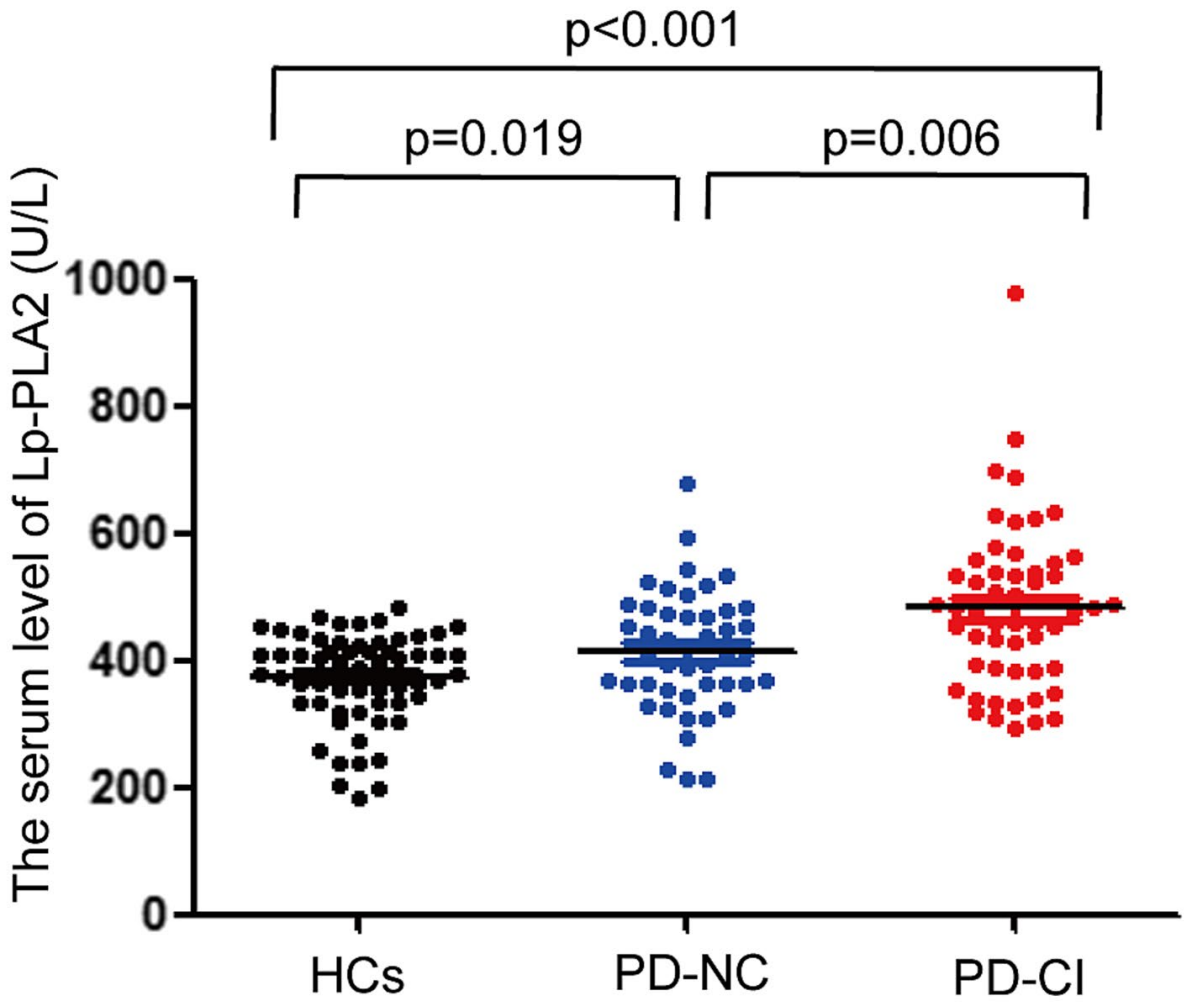
Comparison of Lp-PLA2 level among PD-NC, PD-CI and healthy controls.

The levels of serum Lp-PLA2 activity in PD-NC and PD-CI patients were significantly higher than that in HCs, respectively. In addition, compared with the PD-NC group, the serum Lp-PLA2 activity level was significantly higher in PD-CI patients (*p* < 0.05) (Figure 1).

### 3.4 Correlation analysis between Lp-PLA2 activity and clinical characteristics of PD-CI patients

The correlation between Lp-PLA2 activity and clinical characteristics of PD-CI patients was performed. Our results showed that serum Lp-PLA2 activity level in PD-CI patients was positively correlated with TC (*r* = 0.346, *p* = 0.014), LDL (*r* = 0.382, *p* = 0.006), sdLDL (*r* = 0.349, *p* = 0.010), and ApoB (*r* = 0.449, *p* < 0.001). However, no significant correlations were observed between Lp-PLA2 activity and other laboratory test results (all *p* > 0.05) (Table 3).

**Table 3:** Correlation analysis between Lp-PLA2 activity and clinical characteristics of PD-CI patients.

Variables	Lp-PLA2		Variables	Lp-PLA2	
	*r*	*P*-value		*r*	*P*-value
SBP	0.166	0.235	DBP	0.177	0.205
H&Y stage	−0.124	0.371	BMI	−0.031	0.825
TC	0.346	0.014	TG	0.214	0.135
HDL	0.098	0.498	LDL	0.382	0.006
sdLDL	0.349	0.010	ApoA	0.176	0.202
ApoB	0.449	<0.001	Lp(a)	0.017	0.901
SAA	−0.073	0.598	Glu	0.086	0.554
UA	−0.008	0.958	Cys C	−0.201	0.144
GFR	0.179	0.208	HCY	0.049	0.727

### 3.5 Logistic regression analysis of the risk factors for PD patients with cognitive impairment

A univariate logistic regression analysis was performed to “crudely” explore independent factors that lead to an increased risk of PD-CI, and then multivariate logistic regression was also conducted to further study the risk factors of PD-CI. All variables in Table 4 were entered into the univariate logistic regression model. The results showed that age [odds ratio (OR) 95% confidence interval (CI) = 1.087 (1.042–1.135), *p* < 0.001], Cys C [OR (95%CI) = 23.065 (1.216–437.417), *p* = 0.037], Lp-PLA2 activity [OR (95%CI) = 1.006 (1.002–1.010), *p* = 0.006] were all significantly associated with PD-CI, and higher levels of GFR might be a protective factor for PD-CI [OR (95%CI) = 0.956 (0.926–0.987), *p* = 0.006]. In the multivariate logistic regression analysis in Table 5, we adjusted separately for possible confounders such as age and gender (model 1); age, gender, smoking, drinking (model 2); age, gender, smoking, drinking, hypertension, diabetes, dyslipidemia (model 3). The results revealed that in the above factor-adjusted models, Cys C level was not associated with PD-CI, while the association between Lp-PLA2 activity and the risk of PD-CI was still significantly correlated. In the three models, the OR were 1.008 [(95% CI:1.004–1.013), *p* = 0.001], 1.010 [(95% CI:1.005–1.016), *p* < 0.001] and 1.010 [(95% CI:1.005–1.016), *p* < 0.001], respectively.

**Table 4:** univariate logistic regression analysis of the risk factors for PD patients with cognitive impairment.

	B	S.E.	Walds	*P*-value	OR (95%CI)
Gender	0.219	0.408	0.288	0.592	1.244 (0.560, 2.767)
Age	0.084	0.022	14.830	<0.001	1.087 (1.042, 1.135)
Disease duration	0.013	0.056	0.052	0.820	1.013 (0.908, 1.129)
Family history	0.171	0.842	0.041	0.839	1.186 (0.228, 6.182)
Smoking	1.112	0.639	3.030	0.082	3.041 (0.869, 10.635)
Drinking	1.866	1.115	2.801	0.094	6.463 (0.727, 57.486)
Dyslipidemia	−0.195	0.515	0.143	0.705	0.823 (0.300, 2.259)
Hypertension	−0.003	0.416	0.000	0.993	0.997 (0.441, 2.250)
Diabetes mellitus	−0.565	0.890	0.403	0.525	0.568 (0.099, 3.254)
SBP	0.012	0.011	1.136	0.287	1.012 (0.990, 1.035)
DBP	−0.010	0.019	0.288	0.591	0.990 (0.955, 1.027)
H&Y stage	0.498	0.254	3.857	0.050	1.646 (1.001, 2.706)
BMI	−0.008	0.013	0.374	0.541	0.992 (0.967, 1.018)
TC	−0.022	0.226	0.009	0.924	0.979 (0.629, 1.523)
TG	−0.047	0.279	0.028	0.867	0.954 (0.552, 1.650)
HDL	−0.258	0.605	0.183	0.669	0.772 (0.236, 2.527)
LDL	0.016	0.264	0.004	0.951	1.016 (0.606, 1.706)
sdLDL	−0.153	0.635	0.058	0.809	0.858 (0.247, 2.978)
ApoA	−0.706	0.788	0.804	0.370	0.493 (0.105, 2.312)
ApoB	0.445	0.712	0.392	0.531	1.561 (0.387, 6.296)
Lp(a)	0.007	0.009	0.635	0.426	1.007 (0.989, 1.026)
UA	0.001	0.002	0.305	0.581	1.001 (0.997, 1.006)
Cys C	3.138	1.501	4.370	0.037	23.065 (1.216, 437.417)
GFR	−0.045	0.016	7.516	0.006	0.956 (0.926, 0.987)
Glu	0.392	0.250	2.471	0.116	1.480 (0.908, 2.415)
SAA	0.023	0.015	2.251	0.134	1.023 (0.993, 1.053)
HCY	0.025	0.036	0.483	0.487	1.025 (0.956, 1.099)
Lp-PLA2	0.006	0.002	7.612	0.006	1.006 (1.002, 1.010)

CI, confidence interval; OR, odds ratio.

**Table 5:** multivariate logistic regression analysis of the association between PD patients with cognitive impairment and serum Cys C and Lp-PLA2 activity.

	B	S.E.	Walds	*P*-value	OR (95%CI)
Model 1					
Cys C	1.033	1.645	0.394	0.530	2.809 (0.112, 70.615)
Lp-PLA2	0.008	0.002	11.560	0.001	1.008 (1.004, 1.013)
Model 2					
Cys C	0.707	1.682	0.177	0.674	2.028 (0.075, 54.831)
Lp-PLA2	0.010	0.003	12.932	<0.001	1.010 (1.005, 1.016)
Model 3					
Cys C	0.875	1.769	0.244	0.621	2.398 (0.075, 76.915)
Lp-PLA2	0.010	0.003	12.424	<0.001	1.010 (1.005, 1.016)

CI, confidence interval; OR, odds ratio.

Model 1: adjusting for age, gender; Model 2: adjusting for age, gender, smoking and drinking; Model 3: adjusting for age, gender, smoking, drinking, hypertension, diabetes, dyslipidemia.

### 3.6 Diagnostic value of Lp-PLA2 for identification of parkinsonian cognitive impairment

**Figure 2:**
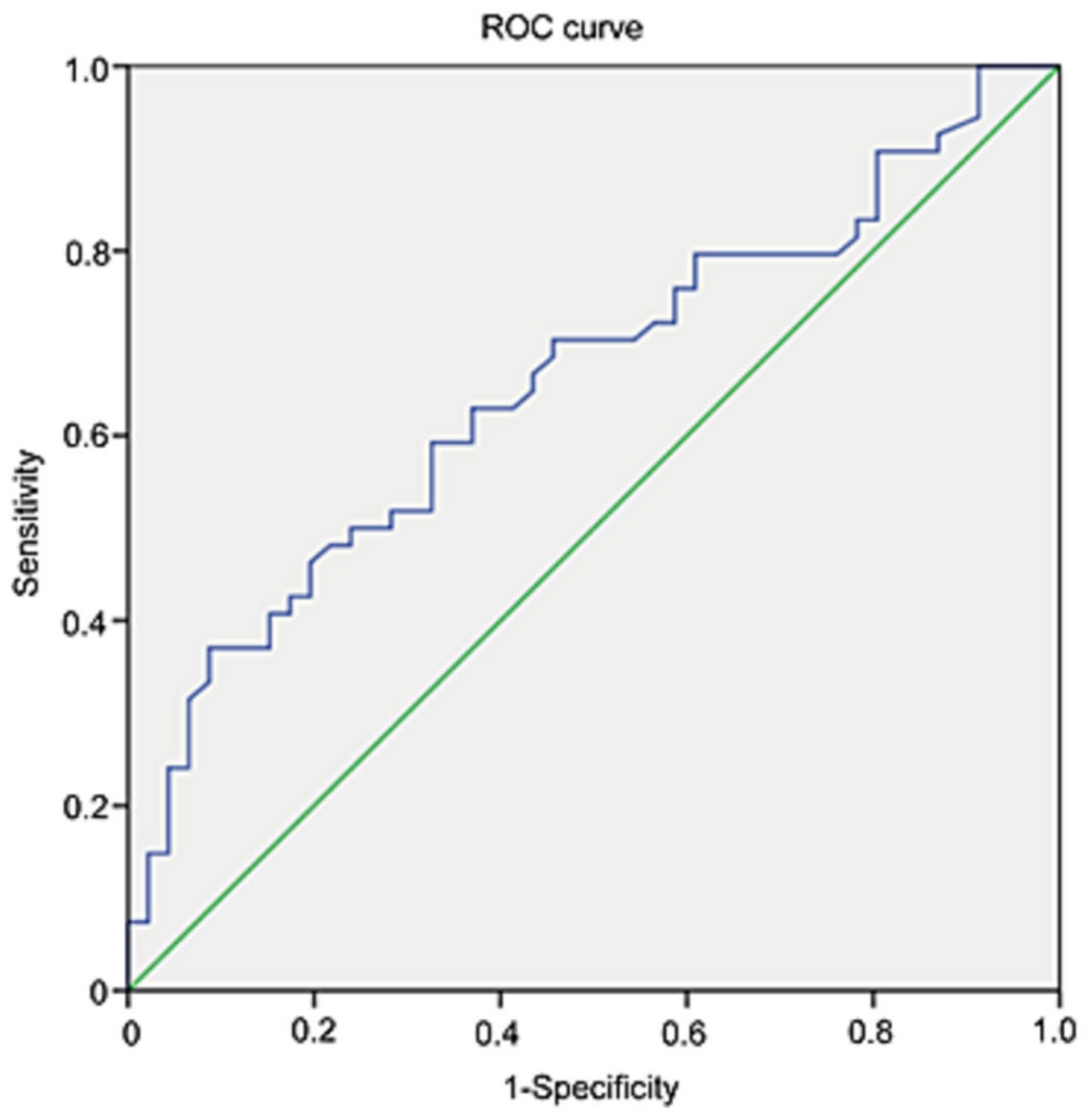
The ROC curve of Lp-PLA2 for discrimination between PD-CI patients and PD-NC patients.

ROC analysis was applied to investigate the diagnostic accuracy of Lp-PLA2 activity in distinguishing PD-CI from PD-NC. The area under the curve of Lp-PLA2 activity for PD-CI diagnosis was 0.659 (95% CI: 0.552–0.765). Based on the ROC curve, the Lp-PLA2 concentration cutoff of 523 U/L was relatively high in specificity (91.3%) and low in sensitivity (37%) for distinguishing between PD-CI group and PD-NC group, with a Youden index of 0.283. The ROC curve of Lp-PLA2 activity for discrimination between the PD-CI group and the PD-NC group was shown in Figure 2.

## 4 Discussion

More and more studies have shown that there are many pathological factors causing cognitive impairment, which may involve oxidative stress, impaired synaptic function, mitochondrial dysfunction, central nervous inflammatory response and other aspects, among which the inflammatory mechanism plays a crucial role (Duggan et al., 2023). Therefore, researchers have been trying to use some relevant biomarkers reflecting vascular and neuroinflammatory damage to diagnose the occurrence of cognitive impairment in the early stage of disease. As an indicator closely related to vascular inflammatory injury, Lp-PLA2 has attracted much attention in cardiovascular and cerebrovascular diseases. Although the roles of Lp-PLA2 in cognitive impairment have been reported, its roles in PD-associated cognitive impairment are still unknown. Continuing our previous research, we collected relevant clinical and laboratory data and conducted a correlation study between Lp-PLA2 and cognitive impairment in patients with Parkinson’s disease. In this investigation, we have observed that individuals with Parkinson’s disease (PD) who experienced cognitive impairment exhibited elevated levels of serum Lp-PLA2 activity in comparison to PD patients with intact cognitive function. Moreover, we have identified a positive correlation between serum Lp-PLA2 activity and TC, LDL, sdLDL, and ApoB. Furthermore, univariate and multivariate logistic regression analyses were employed to demonstrate that increased Lp-PLA2 activity served as an independent risk factor for cognitive impairment in PD. In addition, we have constructed an ROC diagnostic model centered around Lp-PLA2, which has shown promise in aiding the differentiation between cognitively normal PD patients and those experiencing cognitive impairment. Collectively, these findings provide support for the notion that elevated levels of serum Lp-PLA2 activity may serve as a potential predictive biomarker for cognitive impairment in individuals with PD.

Lipoprotein-associated phospholipase A2 (Lp-PLA2), also referred to as platelet-activating factor acetylhydrolase (PAF-AH), is primarily biosynthesized and excreted by macrophages and lymphocytes. Lp-PLA2 mainly produces Lys phosphatidylcholine (lysoPC) and oxidized non-esterified fatty acids (oxNEFAs) by hydrolyzing oxidized LDL (ox-LDL) to exert pro-inflammatory effects, thereby participating in the occurrence and development of atherosclerosis, as well as plaque stability and plaque rupture (Huang et al., 2020). Therefore, Lp-PLA2 is regarded as a reliable marker reflecting vascular inflammatory injury closely related to atherosclerotic diseases, and has been widely studied in cardiovascular and cerebrovascular diseases. Studies have confirmed that higher level of Lp-PLA2 is an independent predictor of coronary heart disease (Yang et al., 2017), myocardial infarction (Sun et al., 2021), acute cerebral infarction (Tao et al., 2020), and cerebral artery stenosis (Wang et al., 2018), and is also closely related to the patient’s admission severity, later treatment, and prognosis. At present, there are still few studies on the roles of Lp-PLA2 in PD. In one of our earlier studies, we have found that higher level of Lp-PLA2 mass is an independent risk factor in PD patients, which is related to the Hoehn-Yahr (H&Y) stage, disease course, and severity of PD, respectively (Wu et al., 2021). In this study, we observed that the Lp-PLA2 activity in PD patients was also significantly higher than that in normal controls. These results indicate that Lp-PLA2 plays a crucial role in PD. Considering the correlation between Lp-PLA2 and cognitive impairment, we further investigated its relationship with cognitive impairment in PD.

Although some studies have reported the relationship between Lp-PLA2 and cognitive impairment, their conclusions are not consistent. In an earlier study, a small cross-sectional study of 78 AD cases, 59 amnestic mild cognitive impairment cases, and 66 cognitively healthy subjects by Davidson et al. found no significant association between Lp-PLA2 and AD (Davidson et al., 2012). Similar results were also found in the other two researches, which showed no significant correlation between Lp-PLA2 mass or activity and AD (van Himbergen et al., 2012; Savas et al., 2016). However, a recent case–control study revealed that higher Lp-PLA2 was independently associated with AD (Doody et al., 2015). Subjects with higher levels of Lp-PLA2 were almost twice as likely to have AD compared with subjects with Lp-PLA2 levels below the median. In addition, there is also some evidence that Lp-PLA2 is negatively related to cognitive function, and high levels of Lp-PLA2 can contribute to prevent the occurrence of cognitive impairment. Zhu et al. analyzed 87 patients with cerebral small vessel disease (CSVD) and found that Lp-PLA2 was independently associated with cognitive impairment and white matter hyperintensities (WMHs) lesions. The level of Lp-PLA2 mass in CSVD patients with mild or severe cognitive impairment was significantly lower than that in patients with normal cognition. These results may be related to the antioxidant effect of Lp-PLA2 (Zhu et al., 2019). However, a recent study supports the opposite conclusion. Huang et al. also proposed that Lp-PLA2 was an independent risk factor for WMHs, but the level of Lp-PLA2 mass was significantly higher in cognitively impaired CSVD patients than in cognitively normal controls (Huang et al., 2021). Currently, increasing evidence suggest that higher levels of Lp-PLA2 are risk factors for cognitive impairment. In a case–control study, high levels of Lp-PLA2 increased the risk of vascular dementia (VD) and AD, independent of other cardiovascular and inflammatory factors (van Oijen et al., 2006; Doody et al., 2015). In addition, Fitzpatrick et al. conducted a population-based longitudinal study of community residents over the age of 65 years, and the results also confirmed that individuals with elevated Lp-PLA2 mass and activity levels were related to increased risk of dementia at an average of 5.4 years of follow-up. And it was not associated with cardiovascular disease (CVD) and its risk factors. Participants in the highest quartile of Lp-PLA2 mass were 50% more likely to develop dementia than those in the lowest quartile (Fitzpatrick et al., 2014). Jiang et al. performed a large cross-sectional study in a Chinese community population, and the results also showed that elevated level of Lp-PLA2 mass was independently associated with the prevalence of cognitive impairment in Chinese adults (Jiang et al., 2016). Based on the sample size of the study and the duration of follow-up, the conclusion that Lp-PLA2 is regarded as a risk factor for cognitive impairment may be convincing. In addition, Cai et al. also found that the mass and activity of Lp-PLA2 were associated with the risk of mild cognitive impairment in Chinese patients with type 2 diabetes (Cai et al., 2017). Although the above-mentioned multiple studies suggest that Lp-PLA2 may be involved in the pathogenesis of cognitive impairment, the role of Lp-PLA2 in cognitive impairment in PD remains unknown.

Our findings not only indicate an elevation in Lp-PLA2 activity levels in PD patients, but also reveal a significant association between higher Lp-PLA2 activity levels and the risk of cognitive impairment in PD. We first screened for meaningful variables by comparing PD patients with cognitive impairment and cognitively normal patients. Compared with cognitively normal PD patients, we found no statistically significant difference in UA. However, some studies had shown that low levels of UA were associated with cognitive impairment in PD patients, and a decrease in UA levels led to a decrease in antioxidant capacity in PD patients (Pellecchia et al., 2016). But we did not find that in our study. This may be related to the different exclusion criteria. They excluded factors that could possibly modify UA levels in the study, such as treatment with diuretics, non-steroidal anti-inflammatory drugs, or other UA altering agents. In our study, we did not exclude these patients with PD. In addition, we found that Cys C and Lp-PLA2 were significantly elevated in cognitively impaired PD patients compared with cognitively normal PD patients. The elevation of Cys C in PD patients with cognitive impairment was consistent with some researches (Hu et al., 2016). Furthermore, in the univariate logistic regression analysis, both Lp-PLA2 and Cys C were independent risk factors for cognitive impairment in PD. However, after adjustment for different PD risk factors, in the multivariate logistic regression analysis model, only Lp -PLA2 had the value of risk prediction. In order to better distinguish PD patients with cognitive impairment, we further analyzed the diagnostic characteristics of Lp-PLA2 for cognitive impairment in PD using ROC curve, and determined the appropriate diagnostic cut-off value.

Although our findings suggest that higher levels of Lp-PLA2 may be a risk factor for cognitive impairment in PD, its mechanistic roles remain unclear. At present, more evidence supports the formation of cerebrovascular atherosclerosis as important factors in cognitive impairment (Shabir et al., 2018). Given that Lp-PLA2 is involved in the formation and progression of vascular atherosclerosis, we speculate that Lp-PLA2 may lead to cognitive impairment by increasing vascular damage and promoting neurodegeneration. Doody RS et al. did not detect the expression of Lp-PLA2 in the brain tissue of AD patients and controls, but they also suggested that the involvement of Lp-PLA2 in the pathogenesis of AD may be related to its vascular injury (Doody et al., 2015). It has been confirmed that Lp-PLA2 and its main enzyme product lysoPC can participate in diabetic retinopathy by disrupting the blood retinal barrier (BRB) (Canning et al., 2016; Acharya et al., 2017). Based on the structural similarity of the BRB and the blood–brain barrier (BBB), we believe that Lp-PLA2 may also disrupt the BBB, thereby indirectly participating in the development of cognitive impairment in PD. In addition, Lp-PLA2 can also hydrolyze ox-LDL to produce OxNEFA. Therefore, high concentrations of fatty acids make the brain more susceptible to oxidative stress, which may be another important mechanism for triggering cognitive dysfunction (Swomley and Butterfield, 2015). More interesting to us is whether Lp-PLA2 can directly or indirectly participate in the pathogenesis of cognitive impairment in PD by affecting key molecules associated with dementia, such as amyloid β or Tau, which requires further researches to reveal this issue.

Although our study suggests that Lp-PLA2 activity is associated with the risk of cognitive impairment in PD, some limitations remain in this study. First, it is necessary to continue to expand the sample size of PD patients with or without cognitive impairment to further confirm the relationship between Lp-PLA2 and the risk of cognitive impairment in PD. Second, long-term follow-up studies for early PD patients are needed to observe whether subjects with high levels of Lp-PLA2 develop PD cognitive impairment in later follow-up studies. Third, in our study, we do not consider whether the subjects use lipid-lowering drugs. Some PD patients or the elderly population of normal controls may have taken oral lipid-lowering drugs, which may lead to a decrease in Lp-PLA2 levels to a certain extent, but it generally will not have much impact on our conclusions. Fourth, in our study, some PD patients had a history of hypertension, diabetes, and cerebral infarction, which may have a certain effect on Lp-PLA2 levels. However, considering that the distribution of the above factors was not significantly different between patients with cognitive impairment in PD and cognitively normal patients, we believe that the impact of this potential bias on the conclusions is limited. Fifth, MOCA is used to assess cognitive status in PD patients, and this score does not always accurately reflect cognitive function as it is sometimes affected by the subject’s education level. Finally, we only detect the activity of Lp-PLA2, but not its mass. Based on the above problems, whether Lp-PLA2 can be used as a predictive biomarker for the diagnosis of cognitive impairment in PD may require more scientific researches to verify.

## 5 Conclusion

In conclusion, our study shows that higher levels of Lp-PLA2 activity in PD patients are associated with the risk of developing cognitive impairment. Therefore, given the wide availability, safety, and convenience of monitoring serum Lp-PLA2 activity, it may serve as a predictive biomarker for cognitive impairment in PD patients.

## Data availability statement

The original contributions presented in the study are included in the article/supplementary material, further inquiries can be directed to the corresponding authors.

## Ethics statement

The studies involving humans were approved by the ethics committee at Tongji Medical College, Huazhong University of Science and Technology. The studies were conducted in accordance with the local legislation and institutional requirements. The participants provided their written informed consent to participate in this study.

## Author contributions

ZW: Funding acquisition, Project administration, Writing – original draft, Writing – review & editing. DS: Software, Writing – original draft, Writing – review & editing. SW: Data curation, Writing – original draft. PC: Methodology, Supervision, Validation, Writing – review & editing. TL: Formal analysis, Investigation, Methodology, Project administration, Writing – original draft, Writing – review & editing.

## References

[ref1] Aarsland D. Batzu L. Halliday G. M. Geurtsen G. J. Ballard C. Ray Chaudhuri K.. (2021). Parkinson disease-associated cognitive impairment. Nat. Rev. Dis. Primers 7:47. doi: 10.1038/s41572-021-00280-3 34210995

[ref2] Acharya N. K. Qi X. Goldwaser E. L. Godsey G. A. Wu H. Kosciuk M. C.. (2017). Retinal pathology is associated with increased blood-retina barrier permeability in a diabetic and hypercholesterolaemic pig model: beneficial effects of the LpPLA(2) inhibitor Darapladib. Diab. Vasc. Dis. Res. 14, 200–213. doi: 10.1177/1479164116683149, PMID: 28301218

[ref3] Bloem B. R. Okun M. S. Klein C. (2021). Parkinson's disease. Lancet 397, 2284–2303. doi: 10.1016/S0140-6736(21)00218-X 33848468

[ref4] Cai R. Huang R. Han J. Sun H. Sun J. Xia W.. (2017). Lipoprotein-associated phospholipase A2 is associated with risk of mild cognitive impairment in Chinese patients with type 2 diabetes. Sci. Rep. 7:12311. doi: 10.1038/s41598-017-12515-z, PMID: 28951620 PMC5615059

[ref5] Canning P. Kenny B. A. Prise V. Glenn J. Sarker M. H. Hudson N.. (2016). Lipoprotein-associated phospholipase A2 (Lp-PLA2) as a therapeutic target to prevent retinal vasopermeability during diabetes. Proc. Natl. Acad. Sci. USA 113, 7213–7218. doi: 10.1073/pnas.1514213113, PMID: 27298369 PMC4932924

[ref6] Davidson J. E. Lockhart A. Amos L. Stirnadel-Farrant H. A. Mooser V. Sollberger M.. (2012). Plasma lipoprotein-associated phospholipase A2 activity in Alzheimer's disease, amnestic mild cognitive impairment, and cognitively healthy elderly subjects: a cross-sectional study. Alzheimers Res. Ther. 4:51. doi: 10.1186/alzrt154, PMID: 23217243 PMC3580460

[ref7] Doody R. S. Demirovic J. Ballantyne C. M. Chan W. Barber R. Powell S.. (2015). Lipoprotein-associated phospholipase A2, homocysteine, and Alzheimer's disease. Alzheimers Dement 1, 464–471. doi: 10.1016/j.dadm.2015.08.001, PMID: 27239525 PMC4879494

[ref8] Duggan M. R. Butler L. Peng Z. Daya G. N. Moghekar A. An Y.. (2023). Plasma proteins related to inflammatory diet predict future cognitive impairment. Mol. Psychiatry 28, 1599–1609. doi: 10.1038/s41380-023-01975-7, PMID: 36737481 PMC10208977

[ref9] Feng F. Chen Y. Wang G. Huang P. Zhu Q. Zhou B. (2022). Correlation of serum CysC, IMA, and Lp-PLA2 levels with type 2 diabetes mellitus patients with lower extremity atherosclerotic occlusive disease. Front. Surg. 9:846470. doi: 10.3389/fsurg.2022.846470, PMID: 35356504 PMC8959309

[ref10] Fitzpatrick A. L. Irizarry M. C. Cushman M. Jenny N. S. Chi G. C. Koro C. (2014). Lipoprotein-associated phospholipase A2 and risk of dementia in the cardiovascular health study. Atherosclerosis 235, 384–391. doi: 10.1016/j.atherosclerosis.2014.04.032, PMID: 24929287 PMC4096578

[ref11] Hu W. D. Chen J. Mao C. J. Feng P. Yang Y. P. Luo W. F.. (2016). Elevated cystatin C levels are associated with cognitive impairment and progression of Parkinson disease. Cogn. Behav. Neurol. 29, 144–149. doi: 10.1097/WNN.0000000000000100, PMID: 27662452

[ref12] Huang F. Wang K. Shen J. (2020). Lipoprotein-associated phospholipase A2: the story continues. Med. Res. Rev. 40, 79–134. doi: 10.1002/med.21597, PMID: 31140638 PMC6973114

[ref13] Huang C. J. Zhou X. Yuan X. Zhang W. Li M. X. You M. Z.. (2021). Contribution of inflammation and Hypoperfusion to white matter Hyperintensities-related cognitive impairment. Front. Neurol. 12:786840. doi: 10.3389/fneur.2021.786840 35058875 PMC8763977

[ref9001] Hughes A. J. Daniel S. E. Lees A. J. (2001). Improved accuracy of clinicaldiagnosis of Lewy body Parkinson’s disease. Neurology. 57, 1497–1499. doi: 10.1212/wnl.57.8.1497 11673599

[ref14] Jiang R. Chen S. Shen Y. Wu J. Chen S. Wang A.. (2016). Higher levels of lipoprotein associated phospholipase A2 is associated with increased prevalence of cognitive impairment: the APAC study. Sci. Rep. 6:33073. doi: 10.1038/srep33073 27609335 PMC5017024

[ref15] Liu L. Zhang X. Jiang N. Liu Y. Wang Q. Jiang G.. (2023). Plasma lipoprotein-associated phospholipase A2 affects cognitive impairment in patients with cerebral microbleeds. Neuropsychiatr. Dis. Treat. 19, 635–646. doi: 10.2147/NDT.S401603, PMID: 36987525 PMC10040165

[ref9002] Pellecchia M. T. Savastano R. Moccia M. Picillo M. Siano P. Erro R.. (2016). Lower serum uric acid is associated with mild cognitive impairment in early Parkinson’s disease: a 4-year follow-up study. J Neural Transm (Vienna) 123, 1399–1402. doi: 10.1007/s00702-016-1622-6, PMID: 27682634

[ref16] Pokharel Y. Mouhanna F. Nambi V. Virani S. S. Hoogeveen R. Alonso A.. (2019). ApoB, small-dense LDL-C, Lp(a), LpPLA(2) activity, and cognitive change. Neurology 92, e2580–e2593. doi: 10.1212/WNL.0000000000007574, PMID: 31043469 PMC6556082

[ref17] Ridker P. M. Rifai N. Macfadyen J. Glynn R. J. Jiao L. Steg P. G.. (2022). Effects of randomized treatment with Icosapent ethyl and a mineral oil comparator on interleukin-1beta, Interleukin-6, C-reactive protein, oxidized low-density lipoprotein cholesterol, homocysteine, lipoprotein(a), and lipoprotein-associated phospholipase A2: a REDUCE-IT biomarker substudy. Circulation 146, 372–379. doi: 10.1161/CIRCULATIONAHA.122.059410, PMID: 35762321

[ref18] Savas S. Kabaroglu C. Alpman A. Sarac F. Yalcin M. A. Parildar Z.. (2016). No relationship between lipoprotein-associated phospholipase A2, proinflammatory cytokines, and neopterin in Alzheimer's disease. Exp. Gerontol. 77, 1–6. doi: 10.1016/j.exger.2016.01.014, PMID: 26828804

[ref19] Shabir O. Berwick J. Francis S. E. (2018). Neurovascular dysfunction in vascular dementia, Alzheimer's and atherosclerosis. BMC Neurosci. 19:62. doi: 10.1186/s12868-018-0465-5, PMID: 30333009 PMC6192291

[ref20] Sun L. Zhu Z. Shi M. Jia Y. Yang P. Wang Y.. (2021). Causal effect of lipoprotein-associated phospholipase A2 activity on coronary artery disease and myocardial infarction: a two-sample Mendelian randomization study. Clin. Chim. Acta 523, 491–496. doi: 10.1016/j.cca.2021.10.039, PMID: 34740601

[ref21] Swomley A. M. Butterfield D. A. (2015). Oxidative stress in Alzheimer disease and mild cognitive impairment: evidence from human data provided by redox proteomics. Arch. Toxicol. 89, 1669–1680. doi: 10.1007/s00204-015-1556-z 26126631

[ref22] Tao L. Shichuan W. Detai Z. Lihua H. (2020). Evaluation of lipoprotein-associated phospholipase A2, serum amyloid a, and fibrinogen as diagnostic biomarkers for patients with acute cerebral infarction. J. Clin. Lab. Anal. 34:e23084. doi: 10.1002/jcla.23084, PMID: 31713292 PMC7083405

[ref23] Van Himbergen T. M. Beiser A. S. Ai M. Seshadri S. Otokozawa S. Au R.. (2012). Biomarkers for insulin resistance and inflammation and the risk for all-cause dementia and alzheimer disease: results from the Framingham heart study. Arch. Neurol. 69, 594–600. doi: 10.1001/archneurol.2011.670, PMID: 22213409 PMC3512190

[ref24] Van Oijen M. Van Der Meer I. M. Hofman A. Witteman J. C. Koudstaal P. J. Breteler M. M. (2006). Lipoprotein-associated phospholipase A2 is associated with risk of dementia. Ann. Neurol. 59, 139–144. doi: 10.1002/ana.20721, PMID: 16278861

[ref25] Wallace E. R. Segerstrom S. C. Van Horne C. G. Schmitt F. A. Koehl L. M. (2022). Meta-analysis of cognition in Parkinson's disease mild cognitive impairment and dementia progression. Neuropsychol. Rev. 32, 149–160. doi: 10.1007/s11065-021-09502-7, PMID: 33860906

[ref26] Wang Y. Zhou B. Zhou P. Yao Y. Cui Q. Liu Y.. (2018). Association of lipoprotein-associated phospholipase A2 mass with asymptomatic cerebral artery stenosis. J. Cell. Mol. Med. 22, 2329–2336. doi: 10.1111/jcmm.13521, PMID: 29424477 PMC5867129

[ref27] Weintraub D. Aarsland D. Biundo R. Dobkin R. Goldman J. Lewis S. (2022). Management of psychiatric and cognitive complications in Parkinson's disease. BMJ 379:e068718. doi: 10.1136/bmj-2021-068718 36280256

[ref28] Wu Z. Wu S. Liang T. Wang L. (2021). Lipoprotein-associated phospholipase A2 is a risk factor for patients with Parkinson's disease. Front. Neurosci. 15:633022. doi: 10.3389/fnins.2021.633022, PMID: 33958981 PMC8093434

[ref29] Yang L. Liu Y. Wang S. Liu T. Cong H. (2017). Association between Lp-PLA2 and coronary heart disease in Chinese patients. J. Int. Med. Res. 45, 159–169. doi: 10.1177/0300060516678145, PMID: 28222638 PMC5536613

[ref30] Ye H. Robak L. A. Yu M. Cykowski M. Shulman J. M. (2023). Genetics and pathogenesis of Parkinson's syndrome. Annu. Rev. Pathol. 18, 95–121. doi: 10.1146/annurev-pathmechdis-031521-034145, PMID: 36100231 PMC10290758

[ref31] Zaman V. Shields D. C. Shams R. Drasites K. P. Matzelle D. Haque A.. (2021). Cellular and molecular pathophysiology in the progression of Parkinson's disease. Metab. Brain Dis. 36, 815–827. doi: 10.1007/s11011-021-00689-5, PMID: 33599945 PMC8170715

[ref32] Zhu S. Wei X. Yang X. Huang Z. Chang Z. Xie F.. (2019). Plasma lipoprotein-associated phospholipase A2 and superoxide dismutase are independent predicators of cognitive impairment in cerebral small vessel disease patients: diagnosis and assessment. Aging Dis. 10, 834–846. doi: 10.14336/AD.2019.0304, PMID: 31440388 PMC6675532

